# School Labor and Bodily Development

**Published:** 1898-06

**Authors:** 


### School Labor and Bodily Development.

Panly (Lubeck) discussed the influence of school labor on health and bodily development at the International Medical Congress in Moscow. According to the report in the *Deutch. Medic. Wochenschr.* he pointed out that the so-called school diseases were due not only to the school but to the home studies (piano-playing, sewing, knitting, painting). In many cases the occurrence of myopia and other disturbances could be traced to defects in the plan of study. We should condemn the slanting writing as taught at present, with an oblique position on the right of the copy-book. Myopia increases with the amount of work done (1 percent in the village school, 10 percent in the higher school, and 28 percent in the gymnasium). According to the investigations of Burgstein, an hour is too long at a time to give instructions to younger children. According to Stresbach and Friedrich, a recess of two hours for dinner is not sufficient time to allow the scholars to recuperate from their morning studies. Therefore recesses of sufficient length, athletics, or, during one or two free afternoons, gymnastic exercises of all kinds, for example, cycling, swimming, and skating, are absolutely necessary. Vacation should not be burdened with vacation exercises. Every school should be under the supervision of a physician. Besides this, it is most urgently recommended to have the teachers instructed in school hygiene.—*Der Kinderarszt*, 1897, *viii.*, 237.

